# Hierarchical ZIF-8 toward Immobilizing *Burkholderia cepacia* Lipase for Application in Biodiesel Preparation

**DOI:** 10.3390/ijms19051424

**Published:** 2018-05-10

**Authors:** Miaad Adnan, Kai Li, Jianhua Wang, Li Xu, Yunjun Yan

**Affiliations:** 1 Key Laboratory of Molecular Biophysics of the Ministry of Education, College of Life Science and Technology, Huazhong University of Science and Technology, Wuhan 430074, China; miaadadnan14@gmail.com (M.A.); D201577434@hust.edu.cn (K.L.); jhwang@hust.edu.cn (J.W.); xuli@hust.edu.cn (L.X.); 2 Ministry of Science and Technology, Baghdad 10001, Iraq

**Keywords:** adsorption method, biodiesel production, *Burkholderia cepacia* lipase (BCL), hexahedral ZIF-8, hierarchical ZIF-8, surfactant

## Abstract

A hierarchical mesoporous zeolitic imidazolate framework (ZIF-8) was processed based on cetyltrimethylammonium bromide (CTAB) as a morphological regulating agent and amino acid (l-histidine) as assisting template agent. *Burkholderia cepacia* lipase (BCL) was successfully immobilized by ZIF-8 as the carrier via an adsorption method (BCL-ZIF-8). The immobilized lipase (BCL) showed utmost activity recovery up to 1279%, a 12-fold boost in its free counterpart. BCL-ZIF-8 was used as a biocatalyst in the transesterification reaction for the production of biodiesel with 93.4% yield. There was no significant lowering of conversion yield relative to original activity for BCL-ZIF-8 when continuously reused for eight cycles. This work provides a new outlook for biotechnological importance by immobilizing lipase on the hybrid catalyst (ZIF-8) and opens the door for its uses in the industrial field.

## 1. Introduction

In light of global industrial acceleration, the need for new energy sources has increased. Substitute fuels for internal combustion engines have attracted significant attention due to the ever-decreasing world petroleum reserves and aggravated global warming [1]. Biodiesel is represented as monoalkyl fatty acid esters from natural and renewable sources, such as animal and vegetable fats [2]. It is manufactured by a chemical (acidic or alkaline) catalyst that gives a high conversion rate in a short reaction time. However, the traditional chemical path has numerous drawbacks, such as enormous energy requirements, difficulties recovery of the catalyst, and prospective environmental pollution [3]. Lipases (E.C.3.1.1.3) are the most widely employed enzymes in biocatalysis, both at an industrial and an academic level. Lipases are capable of catalyzing a variety of reactions such as hydrolysis, alcoholysis, esterification, and transesterification. The synthesizing of biodiesel via lipase catalysts could overcome some of the drawbacks of chemical issues. Moreover, lipase catalysts possess many advantages: they produce high-purity products, are environmentally friendly, decrease energy consumption, and are compatible with a wide range of crude oils, particularly those containing a high amount of fatty acids [4]. In spite of the advantages of lipases, their high cost remains the main barrier to their commercial use in biodiesel [5]. The immobilization of lipase is perfect for the high cost of lipases. The immobilization strategy improves their operational stability, storage time, reusability, and the easy recovery of enzymes [6].

In respect to the immobilization of lipase, numerous studies have been conducted. The adsorption method is easy to perform, but the connection between enzyme and carrier is commonly weak, attributed to the use of a nonporous support such as aluminum oxide, celite, or ion exchange resins, leading to the leaching of lipases [7]. On the other hand, covalent bonds strongly link enzymes to materials, but require many chemical actions and long time periods, resulting in a loss of enzyme activity [8], with carriers like mesoporous silica, chitosan, and rice straw [9]. Entrapment is used to keep enzymes inside beads or polymers and protect them from harsh media, but this suffers from mass transfer and leads to change or loss of enzyme activity, supports such as sol-gel and calcium alginate [10]. To improve immobilization with the adsorption method, a coordinated matrix of carriers is needed, with mesoporous and average surface area allowing the substrates to pass through the pores. They are tiny enough to avert or reduce the leaching of enzymes.

For this purpose, we selected metal-organic frameworks (MOFs) originating from organic and inorganic ingredients. In recent years, these have attracted attention as a new generation of hosts for biocatalyst materials.

Zeolitic imidazolate frameworks (ZIFs), a subclass of MOFs, are a sort of microporous crystal formed by the combination of metal ions Zn^2+^ or Co^2+^ and 2-methylimidazole. ZIF-8 has the benefits of higher porosity, tunable surface features, unusual thermal and chemical stability, negligible cytotoxicity, and easy construction under room temperature. Due to these features, ZIF-8 has attracted considerable interest for use in a wide range of applications such as optics, adsorption, capturing target materials, gas separation [11], chemical sensors [12], drug delivery [13], and catalysis [14].

A strategy directed at surfactant template for synthesis has been selected for manufacturing hierarchical micro-/mesoporous MOFs. For instance, Wu et al. [15] constructed hierarchical ZIF-8 with 5–20 nm pore sizes utilizing cetyltrimethylammonium bromide (CTAB) as a morphological-regulating agent and histidine (His) as aiding factor. Hierarchical ZIF-8 including micro- and mesoporosity has been synthesized in *n*-butylamine in methanol [16]. Qiu et al. [17] obtained micro-/mesoporous MOF ([Cu_3_ (BTC)_2_ (H_2_O)_3_]) by employing (CTAB) as a template-regulating agent and 1,3,5-trimethylbenzene (TMB) as assisting template agent.

In this research, mesoporous hierarchical ZIF-8 was chosen as a carrier for immobilization of *Burkholderia cepacia* lipase (BCL) via the adsorption technique. Several distinct characteristics of immobilized BCL in pores of hierarchical ZIF-8, including optimal conditions for immobilized lipase and morphological properties, were further studied. To conclude, the intended immobilized BCL was used to catalyze the transesterification in the production of biodiesel.

## 2. Results and Discussion

### 2.1. Characterization and Synthesis of Hexahedral, Hierarchical ZIF-8, and Adsorbed Lipase into Mesoporous ZIF-8 (BCL-ZIF-8)

**Figure 1:**
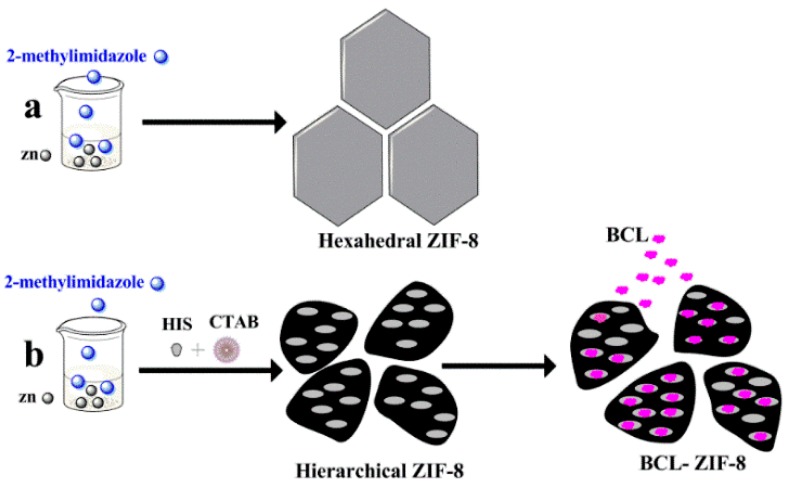
Schematic model to illustrate (**a**) synthesis hexahedral zeolitic imidazolate framework (ZIF-8); (**b**) synthesis hierarchical ZIF-8 and immobilization *Burkholderia cepacia* lipase (BCL)-ZIF-8.

**Figure 2:**
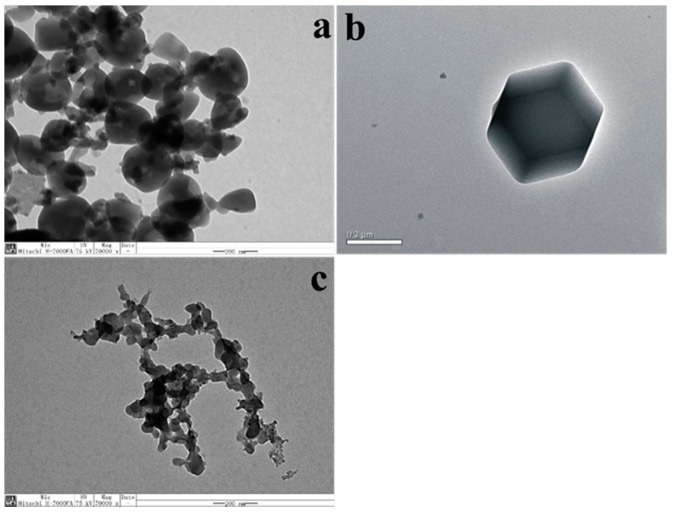
TEM images: (**a**) hierarchical ZIF-8; (**b**) hexahedral ZIF-8; (**c**) BCL-ZIF-8.

**Figure 3:**
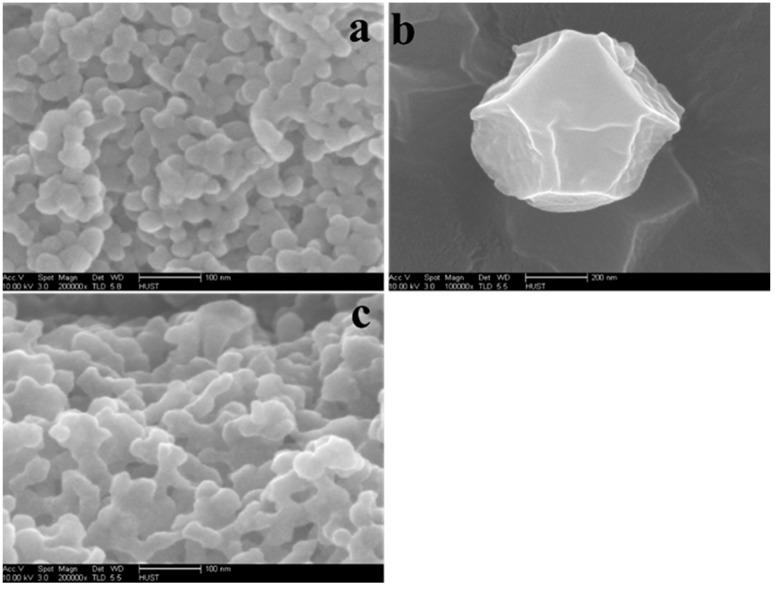
SEM images: (**a**) hierarchical ZIF-8; (**b**) hexahedral ZIF-8; (**c**) BCL-ZIF-8.

Hexahedral and hierarchical micro-/mesoporous ZIF-8 were synthesized and vacuum dried. Then BCL was immobilized on hierarchical ZIF-8 (Figure 1). All samples were characterized by Transmission electron microscopy (TEM) and several randomly distributed mesopores on the surface of hierarchical ZIF-8 were identified; on the contrary, we could not identify these pores in hexahedral ZIF-8 as shown in Figure 2a,b. Figure 2c shows hierarchical ZIF-8 after immobilization, the particles resort to conglomeration as a result of the immobilization. The same differences in morphology between hexahedral and hierarchical ZIF-8 and after immobilization are shown by Scanning electron microscope (SEM) (Figure 3a–c).

**Figure 4:**
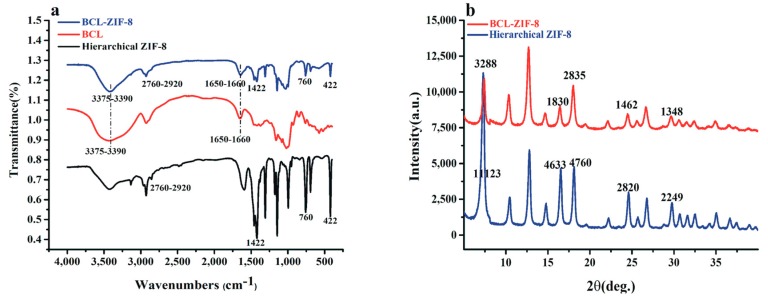
(**a)** Fourier transform infrared (FT-IR) spectra of samples of hierarchical ZIF-8; BCL and BCL-ZIF-8; (**b**) powder X-ray diffraction (PXRD) patterns of samples of hierarchical ZIF-8 and BCL-ZIF-8.

The Fourier transform infrared (FT-IR) spectra of hierarchical ZIF-8, BCL-ZIF-8, and pure BCL are shown in Figure 4a. Bands at 422 cm^−1^ are ascribed to Zn–N. The acute band vibrations at 760 cm^−1^ and 1422 cm^−1^ are attributed to the Hmim ring. Concurrently, the weak peaks at 2760–2920 cm^−1^ were due to the aromatic and aliphatic stretching C–H of Hmim [18]. All of the above mentioned bands were in the spectra of hierarchical ZIF-8 and BCL-ZIF-8. The spectrum for BCL revealed two characteristic absorptions at 1650–1660 and 3375–3390 cm^−1^, which are related to amide (–CO–NH–) I and II bands for protein, respectively [19]. Following the immobilization of BCL in mesoporous ZIF-8, the same characteristic bands of BCL were maintained.

In Figure 4b, Powder X-ray diffraction (PXRD) patterns displayed different peak intensities of hierarchical ZIF-8 and BCL-ZIF-8, respectively. The principal diffraction peaks signify the intensification for ZIF-8 position (2θ) = 7.31°, 16.51°, 18.12°, 24.54°, and 29.74°, which were related to the 11,123, 4633, 4760, 2820, and 2249 as XRD absolute intensity, respectively. In comparison, the intensity of BCL-ZIF-8 was lower owing to the low spacing between the atomic layers in the crystal material [20]. It can therefore be concluded that there was a decrease in ZIF-8 crystallinity after immobilization.

**Figure 5:**
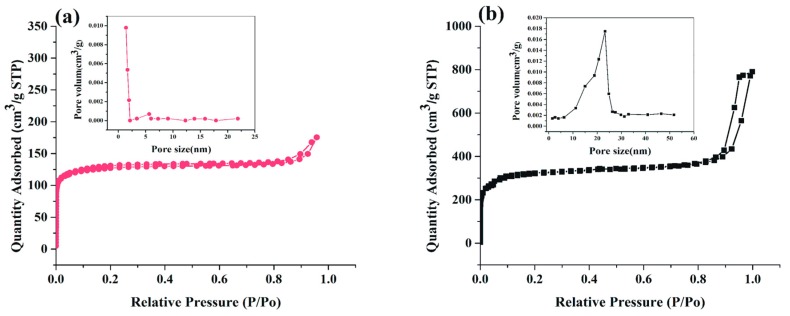
Nitrogen gas adsorption/desorption isotherms and pore size distributions for (**a**) hexahedral ZIF-8 and (**b**) hierarchical ZIF-8.

Figure 5 shows the nitrogen gas adsorption-desorption isotherms and pore size distributions for hexahedral ZIF-8 and hierarchical ZIF-8 specimens. The hexahedral observed in mid-type (I) mode associated with microporous pores was due to the gas adsorption beginning at a lower level of relative pressure and staying at that level. The hierarchical ZIF-8 spotted in type (IV) mode associated with mesoporous pores was due to the gas adsorption starting from a lower level of relative pressure and continuing uptake more than 0.8 P/P0 [21]. The BET specific surface area for the hierarchical ZIF-8 showed to be 751.87 m^2^/g, whereas the specific surface area for hexahedral ZIF-8 was much higher, 1693.21 m^2^/g. Regarding pore size distribution, according to the BJH method from the desorption part, the pore diameter of mesoporous ZIF-8 was, on average, 23.1 nm and hexahedral was 1.4 nm. This difference in surface area and pore size is due to the role of CTAB in forming holes and controlling size during ZIF-8 synthesis.

### 2.2. Effects of the Conditions of Preparation of BCL-ZIF-8 on the Enzyme Activity

As is common, immobilization efficiency and activity recovery were affected by immobilization conditions. This included lipase loading, adsorption time, pH value, and immobilization temperature, which were all studied in this work.

**Figure 6:**
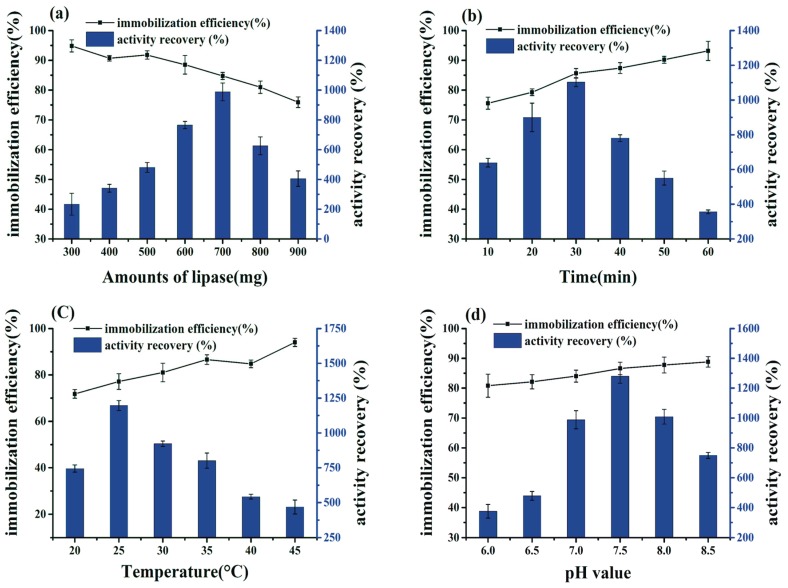
Influence of various immobilization conditions on immobilization efficiency and activity recovery. (**a**) Amounts of lipase; (**b**) adsorption time; (**c**) reaction temperature; (**d**) pH value.

The BCL loading ranged from 300 to 900 mg, increasing by 100 mg each time. The highest activity recovery was 988%, achieved at BCL loading of 700 mg, while immobilization efficiency showed a constant drop whenever loading was raised (Figure 6a). Consequently, 700 mg of BCL loading was chosen as the proper amount of immobilization. A high concentration of BCL loading can lead to an adsorption multilayer on the hydrophobic structure, which decreases the porous diameter and limits diffuse growth [22]. Fan et al. [23] obtained a maximal activity recovery at lipase loading (BCL) of 300 mg, and the immobilization efficiency regularly decreased at values higher or lower than 300 mg. Li et al. [24] got the maximal activity recovery and higher immobilization efficiency when the enzyme loading (BCL) was 3 mg/mg GEAMNP.

As presented in Figure 6b, the immobilization efficiency and enzyme activity increased gradually in the first 30 min of adsorption time. The activity recovery reached a maximum value of 1103% thereafter, declining when the adsorption time exceeded 30 min, whereas immobilization efficiency was relatively settled with a slight increase to 60 min. Thus, 30 min was selected as the ideal adsorption time for BCL immobilization. The watery phase had negative effects on the enzyme activity with increasing adsorption period. This was in accordance with Su et al. [25], who reported enzyme absorption occurring for a long period through immobilization, which might have caused instability in an aqueous phase. Therefore, it is necessary to complete the immobilization procedure within a certain short period of time.

With immobilization temperature between 20 and 45 °C, immobilization efficiency improved continuously. Simultaneously, activity recovery rose in spite of the high temperature, giving a maximal activity recovery of 1196% at 25 °C. After that, activity recovery started to dwindle because of the thermal deactivation of the lipase (Figure 6c). Hence, 25 °C was elected as the best immobilization temperature. The low temperature preserves enzyme properties, including conformation and stability, and inhibits protein denaturation in aqueous buffer, that match well with Liu et al. [26] who reported the increase enzymatic reaction rate with the increase of temperature to a certain level in general, and after that high temperature causes protein denaturation and thus decreases the reaction rate.

pH value is an essential factor that affects enzyme immobilization, as displayed in Figure 6d. An alkaline medium is helpful for immobilization of BCL on ZIF-8. Accordingly, the activity recovery improved gradually to reach the highest value of 1279% when the pH was 7.5. Simultaneously, immobilization efficiency ranged at close levels with a diverse pH value. The probable reason was the difference of pH of the reaction mixture will affect the reactivity of the functional group (i.e., COOH, carboxylic groups) of the enzyme, thereby reducing adsorbing of lipase in ZIF-8 pores [27].

Following these optimal conditions, BCL-ZIF-8 loading of 700 mg, adsorption time 30 min, immobilization temperature 25 °C, and pH value 7.5 led to a 12-fold rise in the activity recovery of an immobilized enzyme compared to a free one. The lipase activity and stability were improved in the following ways. The first factor is related to the immobilization strategy. Adsorption strategy is a physical method that is quick and easy and occurs in mild conditions. Most importantly, the initial structure of the enzyme does not change during immobilization, thus enhancing enzymatic activity. In contrast, a chemical method of immobilization, such as covalent bonding, requires a longer period and higher temperature, and solvents frequently affect the initial structure of the enzyme, leading to a loss of activity [28,29]. Second, as an immobilization support, ZIF-8 has unique characteristics based on its organic/inorganic components, and this leads to the high affinity of the enzyme to the ZIF-8 and its adsorption in mesoporous holes [30,31]. Lipase is the last factor that has a portable component termed as the “lid” which covers the active catalytic center and controls the flow of substrate to the active site of the enzyme. The secondary structure associated with BCL has the potential to be altered by the structure of BCL-ZIF-8. For a period of time, the lid is accessible to the substrate, leading to improvement in lipase activity due to the facility of passage [32,33].

### 2.3. Effect of Transesterification Condition on Biodiesel Production

There is no doubt that kinetic parameters have an impact on biodiesel production. Therefore, a series of investigations was carried out to identify the optimal conditions for the manufacture of fatty acid ethyl esters (FAEEs) by applying the biocatalysts of BCL-ZIF-8 in soybean oil.

**Figure 7:**
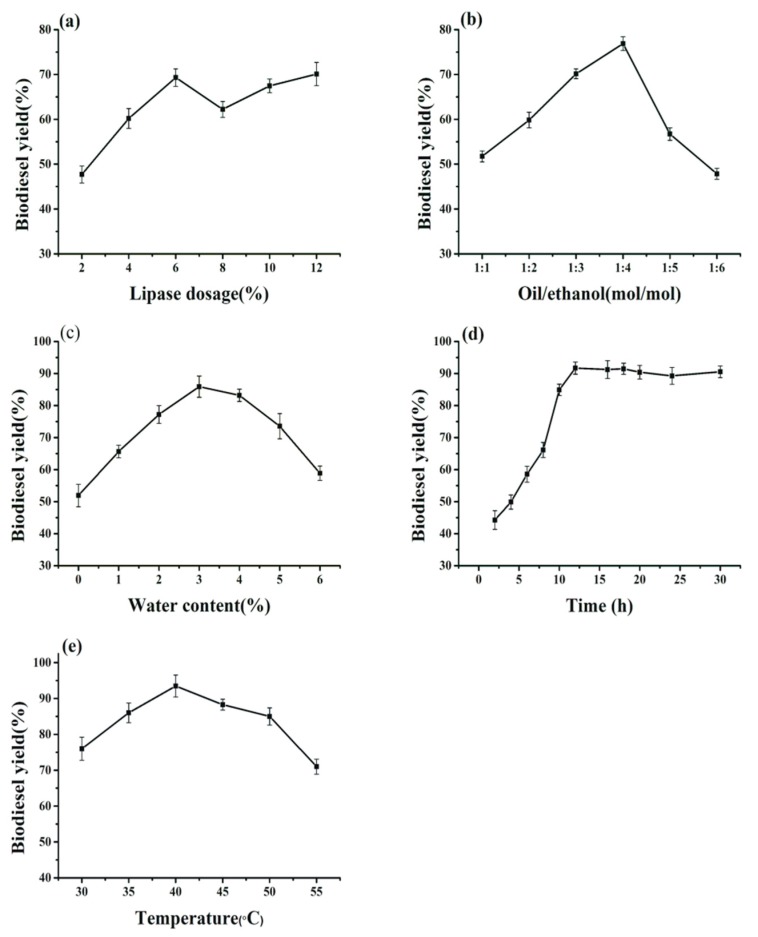
Influences of reaction parameters on biodiesel production catalyzed by BCL-ZIF-8: (**a**) lipase dosage; (**b**) molar ratio of oil to ethanol; (**c**) water content; (**d**) reaction time; (**e**) reaction temperature.

As exhibited in Figure 7a, the output of biodiesel rose by raising the dosage of BCL-ZIF-8 from 2 to 6 wt %, with the highest product of 69.3% found at 6 wt %. There was no significant increase in biodiesel yield when the lipase amount was continuously raised from 6 to 12%. Hence, 6 wt % of immobilized lipase was chosen as the optimal dosage. The biodiesel yield did not increase more, which was attributed to the mass transfer resistance of immobilized BCL and inactivation generated by ethanol [34].

Alcohols have double roles in the transesterification reactions. During the transesterification reactions, an excess of alcohols enhances the reaction rate and shifts the reaction toward elevated production of FAEEs [35,36]. Second, a great concentration of alcohol negatively affects the activity and stability of enzymes by bonding BCL-ZIF-8 with the insoluble alcohol present in the reaction system, leading to a drop in the production of biodiesel [23,37]. Thus, in our experiment, we identified the maximum amount of ethanol added to the reaction that caused the least damage to the immobilized lipase and a maximal yield of fatty acid ethyl esters. The best yield obtained for biodiesel was 76.8%, with a molar ratio of oil to ethanol of 1:4. Meanwhile, the yield started decreasing gradually after the molar ratio is increased to 1:5 and 1:6 (Figure 7b). Rafiei et al. [38] used CRL@ZIF-67 in biodiesel production and achieved the highest conversion when the oil/alcohol molar ratio was 1:6.

Water is regarded as one of the main factors in a mixture of transesterification reaction, because it has the ability to maintain the original conformation of the enzyme and achieve equilibrium in the reaction system for further enhance activity [39,40]. As recorded in Figure 7c, this yield was raised to 85.9% by placed of 3% water to the reaction mixture. Beyond 3%, the biodiesel yield decreased continuously. Though water is important in the reaction, the increased moisture in the system has a negative effect, making the lipase more flexible, resulting in unwanted reactions such as hydrolysis [41]. For example, the maximum biodiesel production for commercial Novozym 435 lipase was obtained without excess of moisture content in a transesterification reaction [42]. The best water content depends on the feedstock oil, lipase type, and immobilized support [43].

In biodiesel production, the time factor and the addition period of alcohol are of great significance. The addition of more than half the amount of alcohol required to the reaction system at the beginning will obstruct the enzyme activity and reduce the biodiesel synthesis [44]. Adnan et al. [6] applied the three-step process with an interval of 8 h to obtain 95.6% output. Liu et al. [45] implemented the three-step approach with an interval of 2 h to obtain 98% yield. Consequently, we adopted a three-step strategy for adding alcohol with time intervals of 2, 4, 6, 8, and 10 h. The maximal yield was 91.7% in 12 h reaction time with intervals 4 h for added ethanol (Figure 7d). We did not see any noticeable improvement in output despite the increased interval to 10 h, which might be due to the dynamic balance that occurred in the components of the reaction [6].

The transesterification reaction is an endothermic reaction, which implies that when the temperature increases, the final output rises. However, enzymes show the best activity at a suitable temperature according to the kind of enzyme and the immobilization technique used. Continually raising the temperature leads to protein denaturation, thus reducing biodiesel synthesis [46]. As noted in Figure 7e, the conversion yield increases with increasing temperature from 25 to 40 °C to the greatest yield of 93.4% at 40 °C. Above 40 °C, the conversion yield gradually diminishes. As stated, the ideal temperature for enzymatic transesterification comes from the interplay between the operational stability of the biocatalyst and the transesterification rate [43]. You et al. [47] obtained a maximal yield of biodiesel at 35 °C when they employed immobilized BCL on modified attapulgite. Li et al. [24] obtained the highest conversion of biodiesel at 45 °C when they used immobilized BCL on heterofunctional magnetic nanoparticles. Thus, the best conditions for transesterification reaction are as follows: BCL-ZIF-8 6 wt %, molar ratio of oil to ethanol 1:4, water content 3%, transesterification time 12 h with three-step addition of alcohol at 4 h intervals, reaction temperature 40 °C, and agitating speed 200 rpm.

### 2.4. Reusability of Immobilized BCL-ZIF-8

**Figure 8:**
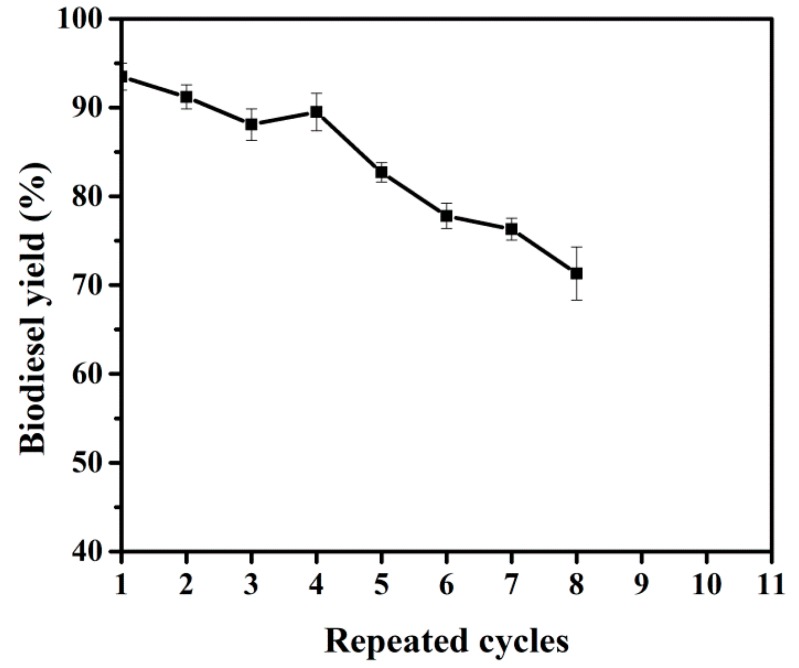
Reuse the catalyzed BCL-ZIF-8 for biodiesel production.

One of the most important reasons to produce immobilized enzymes is their reusability in industrial applications, in addition to achieving high efficiency in industrial products and reducing cost. The reusability of BCL-ZIF-8 in a free solvent system was investigated (Figure 8). BCL-ZIF-8 was kept at 71.3% conversion yield after continuous running for eight cycles. Clearly, the immobilized BCL in the mesoporous ZIF-8 presented higher operational stability. The reported method used a relatively simple design and procedure. The drop rate in biodiesel output with a rising number of cycles was due to the overflow alcohol, the glycerol byproduct forming multilayers around the BCL-ZIF-8. Part of the carrier was broken by the mechanical force resulting from the continuous reaction, leading to enzyme leakage and reduced catalytic activity for the immobilized enzyme [48]. Table 1 illustrates some of the lipases in transesterification reactions for biodiesel production.

## 3. Materials and Methods

### 3.1. Materials

Lipase from BCL was obtained from Sigma Aldrich. The standards of fatty acid methyl/ethyl esters and 2-methylimidazole (HMeIM) were procured from Aladdin Industrial Corporation (Shanghai, China). Coomassie Brilliant Blue G250 and bovine serum albumin (BSA) were obtained from Shenshi Chemical Industry (Wuhan, China). Supplementary reagents were acquired from Sinopharm Chemical Reagent Co., Ltd. (Shanghai, China). Zn (NO_3_)_2_·6H_2_O, Lauric acid, cetyltrimethylammonium bromide (CTAB), triethylamine (TEA), l-histidine (His), acetone, 1-dodecanol, hexane, ethanol, K_2_HPO_4_, KH_2_PO_4_, and sodium hydroxide (NaOH) are in chemical purity. Soybean oil with nearly 99% purity was purchased from local markets. The water used in all the experiments was purified with a water purification system and had a resistivity greater than 18.2 MΩ cm^−1^.

### 3.2. Synthesis of Microporous Hexahedral ZIF-8

According to the method of Zou et al. [59], microporous hexahedral ZIF-8 was synthesized with some modifications. 0.845 g of Zn (NO_3_)_2_.6H_2_O was dissolved in 50 mL deionized (DI) H_2_O and stirred for 5 min until completely dissolved (Solution A). A mixture of 1.85 g 2-methylimidazole and 2.5 g TEA in 50 mL H_2_O for 5 min was prepared, while stirring until completely dissolved (Solution B). Solution B was gradually added to Solution A with continuous stirring at 300 rpm at room temperature until the mixture turned white. Later, it was stirred for 15 min, and ZIF-8 nanoparticles were amassed by centrifugation, then washed with H_2_O three times and finally dried at 80 °C before use.

### 3.3. Synthesis of Mesoporous Hierarchical ZIF-8

Hierarchical ZIF-8 was prepared according to a modified method in the literature [15]: 1.48 g of Zn (NO_3_)_2_·6H_2_O, 1 g of CTAB, and 1.688 g of His were dissolved in 100 mL H_2_O (DI) and stirred until completely dissolved. Later, 100 mL H_2_O were added to a mixture containing 3.24 g 2-methylimidazole and 4 g TEA and stirred until complete dissolution. Then a solution of HMeIM and TEA was mixed slowly with a solution of Zn (NO_3_)_2_.6H_2_O, CTAB, and His at 300 rpm and was kept at room temperature (28 ± 2 °C) for 14 h. The white precipitates were gathered by centrifuged and then washed with water-ethanol solution 50% (*v*/*v*) at 60 °C for 4 h twice to remove unreacted CTAB. Finally, the hierarchical ZIF-8 was washed with phosphate buffer to extract the ethanol and the sample was dried at 100 °C under a vacuum for 10 h for later use.

### 3.4. BCL Immobilization into Mesoporous ZIF-8

Mixing amount of lipase ranging from (300–900 mg) with 0.1 g ZIF-8 in 5 mL phosphate buffer (pH 6–8.5) in a 50 mL tube and sonicated to be redispersed. The mixture was shaken at 200 rpm at a temperature ranging from 20–45 °C for 10–60 min. The immobilized lipases were washed and the supernatant was removed by centrifuging at 8500 rpm at 4 °C for 8 min. Immobilized BCL was dried in a thermostatic vacuum drier for further use. The protein contents of the supernatant and original BCL were measured to define the amount of immobilized enzymes via the Bradford protein assay method with BSA as the standard protein. During the immobilization procedure, the influence of BCL loading, immobilization time, immobilization temperature, and pH value on a specific activity, immobilization efficiency, and lipase activity recovery for the immobilized lipase were studied.

### 3.5. Enzyme Activity Assays

The activity of free and immobilized lipases was tested by an esterification method previously described in literature [60]: a certain amount of the immobilized and free lipase was placed separately in 50 mL capped flasks containing 10 mL of a mixture that contained 1-dodecanol and lauric acid in isooctane at molar ratio 1:1, with 0.01 mL of water. Reactions were performed at 40 °C for 30 min with persistent stirring at 200 rpm. Following this, the reaction was terminated by taking 1 mL of samples and mixing it with 10 mL of ethanol-acetone (1:1, *v*/*v*) as a stop solution. NaOH (0.05 M) was utilized in the titration process to determine remaining acids present in the samples. The one unit for BCL activity (U) was defined as the amount of BCL needed to produce 1 μmol of lauric acid every min under the standard assay system. Immobilization efficiency, specific activity and activity recovery were calculated using Equations (1)–(3) [25]:

(1) $$\mathrm{Immobilization}\;\mathrm{efficiency}\;(\%)\;=\frac{\;\mathrm{immobilized}\;\mathrm{protein}}{\mathrm{total}\;\mathrm{loading}\;\mathrm{protein}}\times \;100\%\;$$

(2) $$\mathrm{Specific}\;\mathrm{activity}\;(U/g\;\mathrm{protein})\;=\frac{\;\mathrm{initial}\;\mathrm{activity}\;}{\mathrm{protein}\;\mathrm{content}\;\mathrm{of}\;\mathrm{immobilized}\;\mathrm{lipase}}$$

(3) $$\mathrm{Activity}\;\mathrm{recovery}\;(\%)\;=\frac{\;\mathrm{activity}\;\mathrm{of}\;\mathrm{immobilized}\;\mathrm{lipase}}{\mathrm{total}\;\mathrm{activity}\;\mathrm{of}\;\mathrm{free}\;\mathrm{lipase}}\times \;100\%\;.$$

### 3.6. Characterization

The specific morphological structure for hexahedral, hierarchical ZIF-8 and BCL-ZIF-8 was determined by a Hitachi SU-8010, field-emission scanning electron microscope (FE-SEM) (Hitachi, Tokyo, Japan). A drop of the samples was placed in the center of the carbon-coated grids to observe the dimensions and details of the samples obtained using a Hitachi H-7000A transmission electron microscope (TEM). FTIR microscopy (Bruker VERTEX 70, Germany) was used in transmission mode utilizing the KBr pellet technique, with a range of 400−4000 cm^−1^. Additionally, powder X-ray diffraction (PXRD) (Empyrean PANalytical Company, Almelo, Netherlands) analyses were carried out using potassium and copper radiation (40 kV, 40 mA) to examine the crystalloid structure of the samples. Nitrogen adsorption–desorption data were recorded using a Micromeritics ASAP 2420 analyzer (Micromeritics Instrument (Shanghai), Norcross, GA, USA) at 77 K. Preceding the measurement, the sample was freed of unwanted or excess gas at 140 °C for 7 h in the line of vacuum. The specific surface area was calculated by applying the Brunauer–Emmett–Teller (BET) method in the domain of relative pressure. The pore-size was determined from the desorption branch of the isotherms using the Barrett–Joyner–Halenda (BJH) method.

### 3.7. Biodiesel Production via Enzymatic Catalysis

The transesterification reaction for biodiesel production was conducted in 50-mL capped flasks stirred at 200 rpm. The mixture was composed of 2.19 g soybean oil, immobilized lipase, water, and ethanol. Ethanol was added in three steps to avoid its inhibitory influence on the immobilized enzyme during the regularly interval. The effects of biodiesel production conditions such as the dosage of BCL-ZIF-8 (2–12 wt % (based on the oil weight, g)), oil-to-alcohol molar ratio from (1:1 to 1:6), water content (1–6 wt % (based on the oil weight, g)), reaction temperature from (30 to 55 °C), and reaction time (2 to 30 h) were methodically probed. The supernatant was collected after a certain reaction time by 4 min of centrifugation at 12,000 rpm. Then, 10 μL of supernatant was added to a mixture of 300 μL of 1.0 mg/mL methyl heptadecanoate as the internal control and 290 μL of *n-*hexane. The mixture was stirred thoroughly for gas chromatography (GC) analysis of biodiesel yield.

### 3.8. Measurement of Biodiesel Yield by GC

GC technique was used to analyze the samples containing fatty acid ethyl esters (FAEEs) according to the same procedure in [25]. The yield of biodiesel was specified using a GC-9790 gas chromatography system (Fuli Analytical Instrument Co., Wenling, China) furnished with Agilent HP-INNOWax capillary column (30 m × 0.25 mm × 0.25 μm; Agilent Technologies, Folsom, CA, USA). The initial temperature of the column was 180 °C; it was then raised to 230 °C at a rate of 3 °C/min and remained at 230 °C for 3 min. The injector and hydrogen flame ionization detector temperatures were set to 230 °C and 280 °C, respectively. The carrier gas used was nitrogen, at a flow rate of 3 mL/min. The injector volume 1 μL using a split mode with flow rate of 50 mL/min (split ratio of 1:25). The biodiesel yield (%) is described as a total of FAEE content in the conversion oil. The product was quantified using Equations (4)–(6) [61]:

(4) $$\mathrm{Weight}\;\mathrm{of}\;\mathrm{FAEE}\;=\;\frac{A_{sample}f_{0}}{A_{internal}W_{internal}}$$

(5) $$f_{0}=\frac{W_{standard}{A\prime }_{internal}}{{W\prime }_{internal}A_{standard}}$$

(6) $$\mathrm{Biodiesel}\;\mathrm{yield}\;(\%)\;=\;\frac{W_{e}}{W_{t}}\;\times \;100\%,$$

where, *A_sample_* is the peak area of the FAEE in the sample, *f_0_* is the response factor, *A_internal_* is the peak area of the internal standard, and *W_internal_* is the weight of the internal standard. *W_standard_* is the weight of standard substance of FAEE, *W'_internal_* is the weight of the internal standard, and *A_standard_* is the peak area of standard substance of FAEE. *W_e_* is the experimental value of all FAEEs identified by the GC equipment and *W_t_* is the theoretical value of all FAEEs.

## 4. Conclusions

A hierarchical micro-/mesoporous ZIF-8 was prepared in aqueous solution using a surfactant (CTAB) as a structure-regulating factor and amino acid (L-histidine) as a co-template. Most importantly, for the first time BCL was successfully immobilized in mesoporous hierarchical ZIF-8 by the adsorption method in a limited time and under standard conditions. Moreover, the esterification of lipase has been improved 12-fold by the immobilization parameters. Lastly, the immobilized BCL was used to produce biodiesel with a high conversion yield and better operational stability. The optimum conditions for biodiesel production were: lipase dosage 6 wt %, molar ratio of oil to ethanol 1:4, water content 3%, transesterification time 12 h with three-step addition of alcohol at 4 h intervals, reaction temperature 40 °C, and agitating speed 200 rpm. The hierarchical BCL-ZIF-8 is promising not only for biofuel production but also for use in industrial applications in the near future.

## Figures and Tables

**Table 1:** Comparison between the BCL-ZIF-8 with different immobilized lipases for biodiesel production.

Enzyme	Substrate	Operating Conditions	System	Acyl Acceptor	Yield (%)	Reusability and Last Yield (%)	References
*Pseudomonas* lipase	Sunflower oil	45 °C; 5 h	Petroleum ether	Methanol	79.0	Non	[49]
*P. fluorescens* lipase	Soybean oil	35 °C; 90 h	Solvent-free	Methanol	80.0	Non	[50]
*Rhizomucor miehei* lipase	Sunflower oil	40 °C; 48 h	Not specified	Methanol	91.2	4 cycle; 67	[51]
*P. expansum* lipase	Corn oil	40 °C; 24 h	Ionic liquids	Methanol	86.0	Non	[52]
*Candida antarctica*	Tallow	45 °C; 36 h	Isopropanol	Methanol	90.0	Non	[53]
*Candida rugosa*	Soybean oil	45 °C; 60 h	Solvent-free	Methanol	78.5	6 cycle; 56	[38]
Novozym 435	Refined palm oil	40 °C; 30 h	Solvent-free	Ethanol	85.0	Non	[54]
*B. cepacia* lipase	Waste cooking oil	40 °C; 35 h	*N*-hexane	Methanol	91.0	5 cycle; 54	[55]
Lipase from *Burkholderia* sp. C20	Olive oil	40 °C; 30 h	Solvent-free	Methanol	92.0	Not specified	[56]
*B. cepacia* lipase	Soybean oil	45 °C;12 h	tert-Butanol	Methanol	96.8	15 cycle; 65	[24]
*B. cepacia* lipase	Jatropha oil	40 °C;12 h	Solvent-free	Methanol	90%	Not specified	[57]
*B. cepacia* lipase	Palm oil	30 °C; 72 h	Solvent-free	Methanol	100.0	10 cycle; 40	[58]
*B. cepacia* lipase	Jatropha oil	35 °C; 24 h	Solvent-free	Methanol	94.0	10 cycle; 89	[47]
*B. cepacia* lipase	Soybean oil	40 °C; 12 h	Solvent-free	Ethanol	93.4	8 cycle; 71.3	This study
